# Triage Errors in Primary and Pre–Primary Care

**DOI:** 10.2196/37209

**Published:** 2022-06-24

**Authors:** Hai Nguyen, Andras Meczner, Krista Burslam-Dawe, Benedict Hayhoe

**Affiliations:** 1 Your.MD Ltd London United Kingdom; 2 Health Services and Population Research Institute of Psychiatry, Psychology and Neuroscience King's College London London United Kingdom; 3 eConsult Ltd London United Kingdom; 4 Department of Primary Care School of Public Health Imperial College London London United Kingdom

**Keywords:** triage errors, pre-primary care, digital symptom checker, primary care, viewpoint, triage, symptom checker, emergency care

## Abstract

Triage errors are a major concern in health care due to resulting harmful delays in treatments or inappropriate allocation of resources. With the increasing popularity of digital symptom checkers in pre–primary care settings, and amid claims that artificial intelligence outperforms doctors, the accuracy of triage by digital symptom checkers is ever more scrutinized. This paper examines the context and challenges of triage in primary care, pre–primary care, and emergency care, as well as reviews existing evidence on the prevalence of triage errors in all three settings. Implications for development, research, and practice are highlighted, and recommendations are made on how digital symptom checkers should be best positioned.

## Introduction

Across health care settings globally, the inability of supply (health care resources) to meet demand (the need of individuals for health care advice) means significant limitations exist on access to medical assessments and treatments. Safe, effective, and fair distribution of health care resources therefore requires some form of filtering and direction, or triage, of individuals within health care services based on type or severity of symptoms and/or initial likely diagnoses.

Emerging health technologies have the potential to provide answers to this problem, in supporting the initial assessment of individuals presenting with symptoms to ensure that they access the right area of the health system with the appropriate degree of urgency. Digital symptom checkers represent one approach, providing users with triage recommendations based on their presenting symptoms and responses to screening questions. However, the extent to which digital symptom checkers can safely be used alongside or in place of existing forms of initial medical assessment is currently unclear, with the potential significance of error in triage recommendation being substantial.

In this article, we discuss existing evidence on triage errors in pre–primary care (using digital symptom checkers), in comparison with primary care and emergency care, and provide recommendations on how digital symptom checkers might be best positioned to support users and existing health systems.

## What Are Triage Errors?

The Oxford English Dictionary defines triage as “the assignment of degrees of urgency of need in order to decide the order of treatment of a large number of injured or ill patients.” The sorting of patients into emergency, urgent, nonurgent, and self-care categories becomes essential in all health care settings where there is a need to manage allocation of limited health care resources [1].

Triage errors can be described as either undertriage or overtriage. Undertriage occurs when the level of urgency of an individual’s condition is underestimated [2] and they are allocated to less urgent health services or treatments than they need, potentially resulting in worsening of their condition. Overtriage refers to inappropriate allocation of health care resources to individuals whose health care needs are less significant [2]. This may lead to unnecessary use of scarce health resources and may also have a direct detrimental impact on affected individuals through unneeded (and potentially harmful) investigations or treatments [3,4].

## Triage in Pre–Primary Care: Context and Challenges

Triage is likely to take place at many stages of a patient’s symptomatic and diagnostic journey, from initial awareness of symptoms through to final established diagnosis and definitive management or resolution of symptoms. Experiencing symptoms is common and frequently does not require medical assessment or treatment [5]. Most individuals will filter and prioritize symptoms that they experience based on factors including personal health beliefs, previous experiences, and informal sources of health information, and seek health care based on the perceived severity of their symptoms/condition, as well as local health system rules, access, and availability.

It has been suggested that the “pre–primary care” health sector, where individuals have reached the stage of considering seeking formal advice on their symptoms but have not yet seen a physician, should be the target of new technological approaches to triage [6]. Building on contemporary interest in self-care, the use of digital technologies to provide detailed and accurate advice and triage provision to support individuals in “self-triage” could enable them to manage their medical problems themselves where possible, or direct them to services of a type and urgency appropriate to their symptoms or condition [7,8].

Digital forms of consultation and triage lack any opportunity for physical examination or for other human interaction, where subtle cues may be picked up. Fully digital consultation systems often lack access to users’ medical histories and are entirely dependent on the data entered by users at the time of consultation. These limitations mean that errors are inevitable. Although face-to-face consultation is often viewed as the gold standard of primary care, it is not free from limitations. These might arise from biases and cultural differences between the clinicians and the patients (for instance, some patients may be reluctant to have blood drawn due to their religious beliefs) [9,10]. To consider the acceptability or otherwise of such errors, it is necessary to understand the extent of error in existing health care triage, both through face-to-face and telephone consultations.

## Existing Evidence on Triage Errors

### Triage Errors in Primary Care

Studies that investigate triage errors in primary care are scarce. A systematic review assessing the safety of telephone triage in out-of-hours care compared with standard face-to-face doctor assessment suggested that triage was safe in 97% (95% CI 96.5%-97.4%) of all patients contacting out-of-hours care and in 89% (95% CI 86.7%-90.2%) of patients with high urgency [11]. This reduced to 46% (95% CI 42.7%-49.8%) when high-risk groups were examined [11]. A triage system in Belgium reported a comparable level of accuracy (98%) when a new French-language algorithm was used [12]. This seems to be consistent with reported rates of triage errors since the 1970s [13]. However, a more recent study in Belgium that compared the triage decisions made by telephone operators and those made by physicians showed a lower level of accuracy [14]. The correctness of the advice given by the operator according to the physicians was 71%, with 12% underestimation of urgency and 17% overestimation [14].

Although some primary care telephone triage is done by doctors, much is done by nurses, sometimes using computer-based clinical decision support systems [15]. A study assessing the safety of telephone triage in general practitioner cooperatives found that triage nurses estimated the level of urgency correctly in 69% of total patients and underestimated the level of urgency in 19% of them [16]. A similar study in the Netherlands reported a comparable rate of triage errors (ie, the level of care was underestimated in 17% of the patients and overestimated in 19%). In Belgium, both the undertriage and overtriage rates were slightly lower, at 10% and 13% of all patients who contacted the out-of-hour telephone service, respectively [17]. In the same study, general practitioners and nurses were found to agree on the level of urgency in 77% of all contacts [17].

### Triage Errors in Emergency Care

In emergency department settings, triage error rates appear to be markedly higher. Tam et al [18] found that triage accuracy in a number of multicentered and single-centered studies was only around 60%, with about 23% of cases undertriaged. A similar rate of triage errors was indicated in a US study, where emergency nurse triage accuracy was recorded for 54% of patients with acute myocardial infarction [19]. Better triage accuracy was recorded in a study in South Korea, where retrospective comparison of records of patients admitted to two emergency departments with a gold standard method (based on a 5-level triage scale reviewed by medical experts) [20] found disagreement in 14.7% of the cases (10% overtriage and 5% undertriage). A comparable 17% triage error rate was reported in a study in Brazil using similar methods [21]. Although triage accuracy varied across studies and there is no standardized acceptable triage rate for all patients, the American College of Surgeons has suggested an acceptable rate of undertriage for trauma patients of 5% and 25%-35% for overtriage [18,22]. It is worth noting that relatively high overtriage rates may be seen in emergency care settings where access to rapid imaging or other investigations allows for subsequent “downgrading” of triage.

### Triage Errors in Digital Symptom Checkers

The accuracy of digital symptom checkers in providing triage has been met with skepticism. There is limited evidence in this area, but vignette studies have suggested that triage error rates have been shown to be high for digital symptom checkers [23,24]. One study compared 12 publicly available symptom checkers and reported that only 51% of triage decisions for the top 5 diagnoses were correct [23]. However, this is the mean rate of errors, which may be skewed by a wide range of triage accuracy between the least and most accurate symptom checkers (22%-72%) [23]. The rates of triage errors increase with condition urgency [23,25]. The level of urgency was found to be appropriately assessed in a small proportion of emergency cases with ophthalmic diagnoses (39%, 95% CI 14%-64%) [26]. When applied in emergency department settings, symptom checkers were reported to be inadequately sensitive to emergency cases, with triage accuracy between 45%-75% of total patients [27-30]. However, in a recent study using digital patient self-triage in a hospital emergency department, a digital tool showed higher sensitivity to high-acuity conditions and similar specificity for low-acuity conditions when compared with standard nurse triage using the Manchester Triage System; it also tended to result in overtriage of patients when compared with standard nurse triage [31].

Triage advice provided by symptom checkers is found to be more risk averse than that provided by health care professionals [30,32], with 85% of the users advised to see their doctor in one study [33]. However, in a 5-year follow-up evaluation study, it was observed that symptom checkers in 2020 are less risk averse (odds of 1.11:1, overtriage errors to undertriage errors) than in 2015 (odds of 2.82:1) [24]. Triage errors in emergencies, nevertheless, are still high, with 40% of emergency cases being missed by symptom checkers [24].

Although most studies regarding the accuracy of symptom checkers were carried out through clinical vignettes [23,24], some clinical trials have been conducted to compare the rates of triage error of face-to-face consultation with a physician and digital symptom assessment technologies [34-37]. Results from these clinical trials show that while symptom checkers did not perform as well as face-to-face consultation, correct triage for certain health conditions was still achieved in a higher proportion of patients than expected [37]. Some symptom checkers were reported to attain a sensitivity level of over 50% [36], consistent with previous findings [23].

Evidence of triage error rates in primary care, in emergency care, and by symptom checkers is summarized in Table 1.

**Table 1:** Triage errors in primary care, in emergency care, and by digital symptom checkers.

	Overtriage	Undertriage
Primary care	13%-19%	10%-19%
Emergency care	10%-35%	5%-23%
Symptom checker	No specific rate of overtriage reported. Mean rate of triage accuracy reported to be around 50%, with a range of 22%-72%	No specific rate of undertriage reported. Mean rate of triage accuracy reported to be around 50%, with a range of 22%-72%

## Discussion

### Summary

Triage error rates in primary and emergency care vary widely across the literature [38], and differing settings and definitions of triage across settings make comparison difficult. The overall level of accuracy of out-of-hour telephone triage was between 69% and 98%. Undertriage rates ranged from 10%-19% in primary care setting and 5%-23% in emergency setting. Overtriage rates ranged from 13%-19% in primary care setting and 10%-35% in emergency setting. Based on limited evidence, digital symptom checkers have relatively low triage accuracy, with a mean error rate of around 50% [25,30]. However, this is likely skewed by outliers caused by the most and least accurate tools, ranging from 22%-72% [23]. Although the errors tend to be over- rather than undertriage, with users advised to visit a doctor in 85% of cases in one study even when symptoms were appropriate for self-care [33], symptom checkers are increasingly less risk averse [24].

### Limitations

It is worth noting that this article is not a formal systematic review, thus no specific strategies or selection criteria were applied to our literature search. This might result in potentially relevant studies being missed, despite our best effort to ensure appropriate studies regarding triage accuracy were included. However, from our consideration of the literature, we observed a high level of heterogeneity among the rates of triage errors across studies. The heterogeneity of triage error rates in primary care, pre–primary care, and emergency care is attributable to a number of factors. Most importantly, case mix and approach to/purpose of triage differ substantially across these settings. The number and type of conditions considered in each study also differed. Although the majority of studies included a mix of acute and chronic conditions, some only considered one type of disease (eg, chronic mental health disorders). Studies that assessed triage accuracy in more conditions were more likely to report higher error rates. In addition, the methods used to identify triage errors were heterogeneous. Eight methods were commonly employed in assessing triage accuracy, namely autopsies, patient and provider surveys, standardized patients, second reviews, diagnostic testing audit, malpractice claims, case reviews, and voluntary reports [3]. Studies that used different methods were found to report significantly different rates of errors [3]. Finally, there appeared to be a lack of clarity in the definition and comparison of triage errors. Some studies did not specify whether the triage errors were overtriage or undertriage. This lack of clarity and consistency means it is not possible to draw conclusions or make clear recommendations as to an acceptable error rate for symptom checkers.

### Implications for Development and Practice

Consideration of triage error in primary care is particularly timely in the current unprecedented public health context. The recent COVID-19 pandemic has challenged the ability of health systems worldwide to meet demand, with services in some countries completely overwhelmed. A pressing need to avoid all but the most urgent and essential health service use, and to limit face-to-face interaction between health care professionals and members of the public to an absolute minimum, has led to the adoption of a “remote total triage” system in primary care using telephone and online consulting in many countries [39].

Whether the digital symptom checkers’ level of performance for triage is acceptable depends on the purposes for which they are used [25]. If symptom checkers are seen as a replacement for seeing physicians, they would currently be an inferior alternative [25]. However, if used by individuals to gather quick and accessible information about particular conditions, they are likely to be superior to self-directed internet searches using online search engines [25]. This is especially appropriate when only the best-performing symptom checkers with low triage error rates are used. It is also worth noting that artificial intelligence technology is constantly improving, potentially making it possible for triage made by digital symptom checkers to become more accurate and thus become a safe and useful addition to traditional face-to-face consultations.

Although seeking to avoid unnecessary burden on health services, the lack of available background information and inability to include information from physical examinations or nonverbal cues means that any remote assessment system will likely need to take a risk averse approach to triage. Thus, it is arguably appropriate that digital triage tools adopt this approach.

### Implications for Research and Development

Most studies assessing the triage error rates among symptom checkers are conducted through clinical vignettes. The preparation and evaluation of vignettes need to be standardized to allow for external validity and comparability. Furthermore, clinical trials where symptom checkers’ rates of triage error are compared with those of face-to-face consultation should be encouraged. This method not only enables the assessment of triage accuracy but also allows the examination of users’ compliance with triage advice and possible benefits for the health care system.

There is little evidence on users’ compliance with triage advice, in either traditional forms of triage or that given by digital symptom checkers, nor is there data on consequences of symptom checker errors. Additionally, little is currently known about patient expectations and health beliefs in relation to digital diagnostic and triage tools. It seems likely that most individuals would place lower weighting on the advice of a symptom checker than a human clinician, and use the information provided by these tools as part of their decision-making process. However, in times of increasing reliance on digital technology, it is possible that some individuals may have greater trust in these tools than might be expected. Research is clearly needed to clarify these questions, but developers should assume a relatively high degree of reliance of users on the recommendations that symptom checkers provide.

In addition, well-conducted research to understand the clinical effectiveness of digital symptom checkers in effectively triaging individuals (ie, offering appropriate self-care advice or assigning to appropriate services) is urgently needed. To inform decisions of users and policy makers adequately, this must incorporate formal comparison with existing provision, with a focus on primary care telephone and online triage.

### Recommendations

1. Digital symptom checkers should largely position themselves in the pre–primary care triage/self-care area—evidence does not currently support the ability of artificial intelligence to provide effective consultations at the level of those that would normally take place in traditional face-to-face primary care or emergency department setting.
2. Digital symptom checkers can be appropriately promoted as a safer alternative/effective addition to existing sources of information (such as self-directed online searches) for individuals prior to seeking formal health advice.
3. Providers of digital symptom checkers should seek to ensure that triage error rates fall within the lower thresholds demonstrated in existing evidence of those in primary care telephone triage (acknowledging the substantial limitations of this literature).
4. Developers of symptom checkers should make efforts to expand the evidence base in this area, including establishing systems to gain user feedback on triage accuracy/appropriateness, as well as engaging with academic partners to carry out formal research. Findings in terms of limitations and error rates should be clearly publicized and highlighted to users.
5. Current methods employed to study symptom checkers’ triage accuracy such as case vignette studies should be standardized to allow for external validity and comparability. Clinical trials where key outcome measures include the accuracy of both outcome conditions and triage should also be conducted. A clear distinction between over- and undertriage should be made to provide data for safety monitoring and economic evaluation.

### Conclusion

There is very limited evidence and no clear gold standard comparison for triage errors in digital symptom checkers, meaning that it is not possible to make recommendations on an acceptable error rate. Positioning symptom checkers in the self-care/pre–primary care triage setting therefore seems to be most appropriate and where they can likely add value for individuals experiencing symptoms. Industry and academics should work together to develop the necessary evidence, and efforts should be made to collect user feedback and outcomes data. Until clearer comparisons with existing care are available, digital symptom checkers and triage tools should appropriately continue to take a risk averse approach in the recommendations they give to users.

## Abbreviations

NHS: National Health Service
NIHR: National Institute for Health and Care Research
